# Prospective changes of cardiac iron and function by MR in pediatric thalassemia major patients treated with different chelators or not chelated

**DOI:** 10.1186/1532-429X-17-S1-P365

**Published:** 2015-02-03

**Authors:** Antonella Meloni, Lorella Pitrolo, Mari Giovanna Neri, Gennaro Restaino, Chiara Tudisca, Paolo Preziosi, Petra Keilberg, Sabrina Armari, Vincenzo Positano, Alessia Pepe

**Affiliations:** 1 CMR Unit, Fondazione G. Monasterio CNR-Regione Toscana, Pisa, Italy; 2 Ematologia II con Talassemia, Ospedale "V. Cervello", Palermo, Italy; 3 Reparto di Pediatria, Azienda Ospedaliera di Legnago, Legnago, Italy; 4 Dipartimento di Radiologia, Un. Cattolica del Sacro Cuore - Centro di Ricerca e Formazione ad Alta Tecnologia "G. Paolo II", Campobasso, Italy; 5 Istituto di Radiologia, Policlinico "Paolo Giaccone", Palermo, Italy; 6 U.O.C. Diagnostica per Immagini e Interventistica, Policlinico "Casilino", Roma, Italy

## Background

There are no prospective studies comparing the effectiveness of the three iron chelators commercially available in preventing or decreasing iron overload in the heart in pediatric thalassemia major (TM) patients. Our aim was to evaluate the changes in cardiac iron and function by quantitative magnetic resonance imaging (MRI) over a follow-up (FU) of 18 months in pediatric TM patients treated with one of the 3 available iron chelators in monotherapy or non-chelated.

## Methods

Among the first 1611 TM patients enrolled in the MIOT (Myocardial Iron Overload in Thalassemia) network, we considered pediatric patients who had maintained the same chelation regimen between the two MRI scans. Myocardial iron overload (MIO) was quantified by a multislice multiecho T2* sequence. Biventricular function parameters were evaluated by cine images. Due to the low sample size, no inter-treatment comparisons were performed.

## Results

Four groups of patients were identified: 6 patients (3 F, 10.0±2.2 years) treated with desferioxamine (DFO- mean dosage 43.7±6.8 mg/kg/die), 7 patients (3 F, 15.5±1.7 yrs) treated with deferiprone (DFP- mean dosage 75.0±9.2 mg/kg/die), 39 patients (13 F, 13.58±3.39 yrs) treated with deferasirox (DFX- mean dosage 26.6±6.7 mg/kg/die), and 2 patients (2 F, 11.1±5.3 yrs) not chelated because they had performed a bone marrow transplantation. Compliance to chelation therapy was excellent/good in all treated groups.

At baseline in DFO, DFP and no chelated groups no patient showed a global heart T2* value<20 ms. In all 4 groups all patients who showed no cardiac iron overload at baseline maintained at the FU the same status. At baseline in DFX group 5 patients had heart T2* values<20 ms. The 4 patients with intermediate cardiac iron (T2* 10-20 ms) at the baseline showed no iron at the FU while the patient with severe cardiac iron (T2*<10 ms) remained in the same status at the FU. Non chelated patients had higher global heart T2* values at baseline (non-chelated 37.7±0.5 ms > DFP 35.3±4.9 ms > DFX 32.7±9.6 ms > DFO 31.9±10.5 ms) while DFP patients had higher global heart T2* values at FU (DFP 39.5±6.1 ms > DFX 34.2±7.3 ms > DFO 33.6±7.9 ms > non-chelated 28.9±4.0 ms).

In the DFO group at baseline 1 patient showed pathological left ventricular ejection fraction (LVEF) and he recovered at the follow up. In the DFP group at baseline 2 patients showed pathological LVEF and both recovered at the follow up. In the DFX group at baseline 3 patients showed pathological LVEF: 2 recovered at the FU and 1 did not perform the evaluation of the cardiac function at FU due to technical reasons. Conversely 9 patients with normal LVEF at baseline showed pathological LVEF at the FU.

## Conclusions

In this young population, DFP and DFO seem to be more effective versus the cardiac iron with a concordant positive effect on the global systolic function. However, further prospective studies are needed on larger study population to confirm the data.

## Funding

The MIOT project receives "no-profit support" from industrial sponsorships (Chiesi Farmaceutici S.p.A. and ApoPharma Inc.).

